# Measuring industrial lumber production using nighttime lights: A focus study on lumber mills in British Columbia, Canada

**DOI:** 10.1371/journal.pone.0273740

**Published:** 2022-09-13

**Authors:** Lukas R. Jarron, Nicholas C. Coops, Dominik Roeser

**Affiliations:** Department of Forest Resources Management, University of British Columbia, Vancouver, B.C., Canada; Universidade Federal de Uberlandia, BRAZIL

## Abstract

Nighttime lights (NTL) are the procurement of remotely sensed artificial illumination from the Visible Infrared Imaging Radiometer Suite (VIIRS) satellite. NTL provides a unique perspective on anthropogenic activity by characterizing spatial and temporal patterns related to economic trends and human development. In this study, we assess the ability of NTL to characterize trends associated with industrial lumber production in British Columbia, Canada. We establish the presence of a logarithmic relationship between NTL and lumber mill production capacity (R^2^ = 0.69–0.82). The ability of NTL to temporally identify mill closures is then demonstrated by differentiating pairs of active and closed mills. We also identify Granger causality and co-integration between NTL and monthly lumber production, highlighting the predictive capability of NTL to forecast production. We then utilize this relationship to build linear regression models that utilize NTL data to estimate monthly (R^2^ = 0.33), quarterly (R^2^ = 0.58), and annual (R^2^ = 0.90) lumber production without reported data.

## Introduction

Anthropogenic activities drive the world economy, influence climate and impact surface conditions on the Earth. However, these activities are not evenly distributed spatially, or in intensity, with variations in anthropogenic activity clearly visible from space. Illumination from anthropogenic activities and population centers have been observed from space since the 1970’s when nighttime satellite images were first used to map oil fields [1], population density [2] and energy consumption [3]. A notable advancement came in 1992 when the Defense Meteorological Satellite Program (DMSP) first acquired nighttime lights (NTL) from its Optical Linescan System (OLS). Since then, NTL has become an earth observation data set used to locate, measure, and compare anthropogentic activities around the globe. While mapping the spatial distribution of NTL has proved useful, a key benefit of the data archive is gaining temporal information on changes in NTL over time. Ongoing data capture increases a dense data archive, allowing historical time-series to be created and NTL fluctuations over time to be compared. This has led to a multitude of studies assessing temporal patterns of NTL around the globe [4–7].

One of the most commonly correlated regional-level economic indicators known to be strongly related to NTL is gross domestic product (GDP) [8–10]. In developed, and rapidly developing countries, socio-economic patterns relating NTL to urbanization [11, 12], population [13], and land cover [14], are also well documented. Studies have confirmed higher levels of GDP are tied to greater energy consumption and urbanization which are then routinely observed using NTL data. At finer spatial scales NTL has been used to map the expansion of cities [10, 15], in-use steel stock [16], fishing boats [17], gas flares [18] and forest fires [19].

Despite the success of these studies, the original DMSP/OLS data has several quality issues. Blooming is particularly problematic [20] which is when pixels are averaged and artificial light from lit pixels spills over into pixels that contain no artificial light, such as water. Saturation presents another challenge where at coarse spatial resolutions, extremely bright pixels are no longer represented equally to their true illumination [21, 22]. In 2011 a NASA and NOAA joint effort satellite was launched carrying the Visible Radiometer Suite (VIIRS) instrument to replace its OLS predecessor providing increased spatial and radiometric resolution (500 m vs 2000 m) and the ability to detect lower levels of illumination [23].

The increased spatial resolution of VIIRS has been shown to allow detection of variable illumination levels based on industrial production [24], and transportation infrastructure [25, 26], and employee populations [27] This highlights the potential for NTL to track “boom and bust” cyclical industries, such as forestry which is highly susceptible to international trade regimes and market conditions. Given that NTL has been utilized to detect production trends in other volatile natural resource-based industries including mining, fishing, and oil and gas [28] it has the potential to be applied to forestry production as well. Bruederle and Hodler [29] demonstrate that VIIRS NTL is indeed a useful proxy for measuring human development factors such as household wealth at a local township scale. These communities rely heavily on industrial forestry for economic well-being [30], therefore the ability to map the conditions related to the forest industry would provide valuable insight into the socio-economic patterns of the communities, forestry companies and province as a whole [31] In British Columbia (B.C.), Canada, the forest industry directly contributes 2.5% to provincial GDP each year and up to 20% of employment in isolated regions [32], providing wealth and employment to many of the province’s smaller communities.

In this study, we assess the ability of NTL to measure changes in lumber production across B.C. Both harvesting and lumber manufacturing play an integral role in the British Columbia economy, especially in smaller communities. Since production of lumber is typically focused around industrial-sized mills in these small communities, NTL should reasonably be able to discern the fluctuations in production at these mills which are likely to be the dominant artificial light generator on these landscapes. Many large lumber mills run night shifts and have import and export shipments running well into the night, which could potentially be detected by NTL data. We first evaluate the direct relationship between mean NTL radiance and estimated lumber production capacity. We then assess the ability of NTL to detect decreases in production associated with recent individual mill closures. Lastly, we assess the ability of NTL to be used as a province-wide proxy for trends relating to the industrial forest sector by comparing long-term lumber production trends to NTL trends over the seven-year monthly time-series. The ability to measure localized lumber production across B.C. using NTL both spatially and temporally will provide insight into community-level GDP and environmental impacts related to the volume of timber being extracted for lumber production.

### The B.C. forest industry

**Fig 1:**
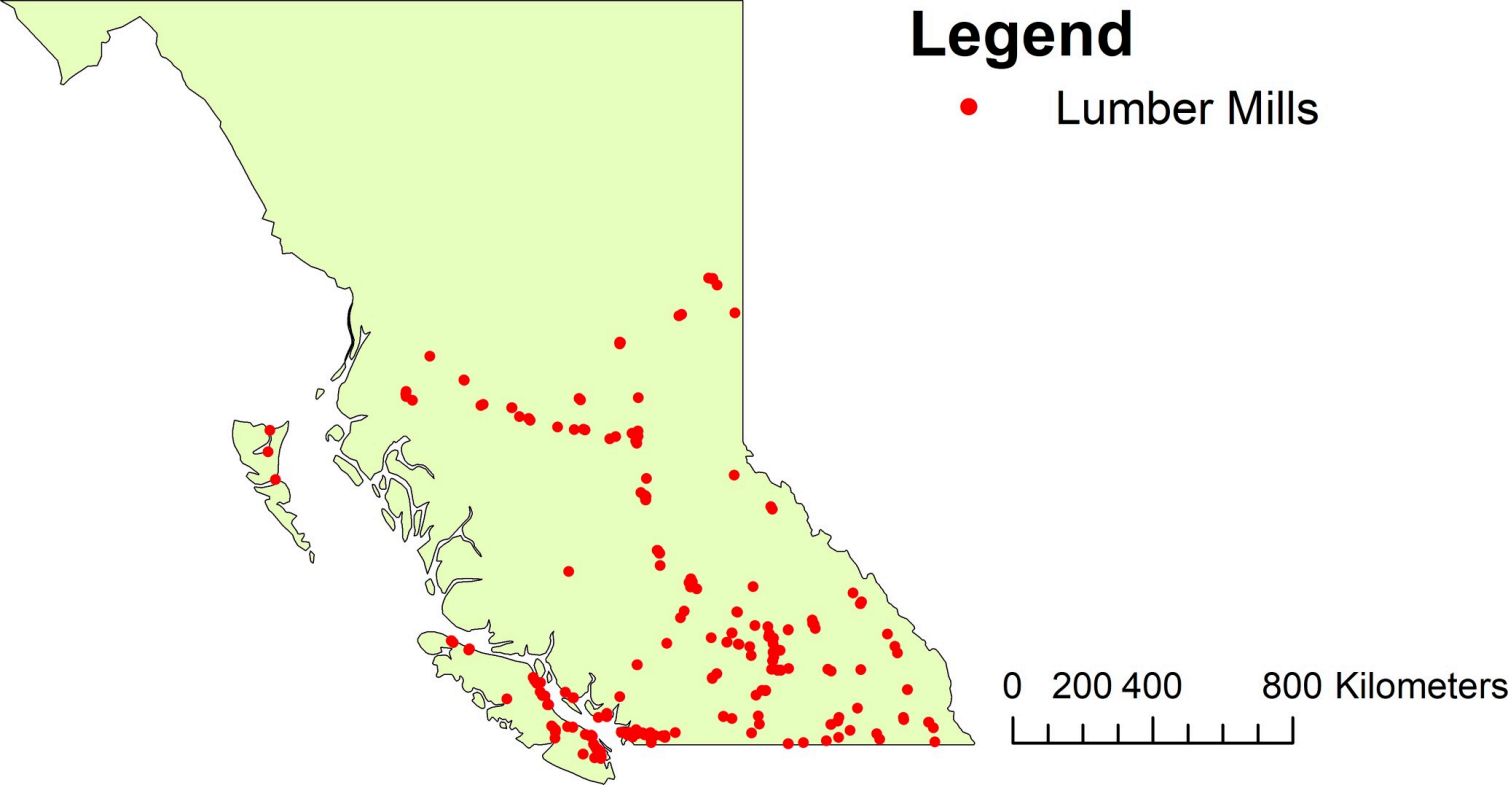
Study area displaying location of all lumber mills in B.C.

British Columbia covers a total of 944,735 km^2^ and a range of topographies and climates. Latitudes span biogeoclimatic regions ranging from northern boreal forest to rolling interior plateaus and wet coastal rainforests. The province is split into three primary natural resource regions consisting of the north interior zone, south interior zone, and coastal zone. Lumber production facility data were extracted from provincial databases as point shapefiles [33] which include the geographic coordinates, operating company and estimated production capacity of each lumber facility. We focused on the largest 80% of lumber processing facilities located across the Province, totaling 122 individual mills. Mills in the north and south zones experience considerable snow in winter months, while the coast zone receives heavy rain and constant cloud cover during winter. Estimated lumber capacity varies by mill and ranges from 0.5 to 473 million boards per year and includes operations from family-owned mills to multi-national corporations. Fig 1 provides a visualization of the lumber mill locations across B.C.

### Satellite data

We combined 84 monthly radiance composites collected from the VIIRS (v1) stray-light corrected dataset spanning a 7-year period from January 2014 to December 2020 [28]. This version is acquired from the Day/Night Band (DNB) sensitive to wavelengths between 0.5–0.9 um, corrected for the impact of lightning, cloud cover, and lunar radiance. NTL radiance values (nW/cm^2^/sr^-1^) have been resampled via spatial averaging from a spatial resolution of 742 m to 500 m, representing the best available resolution for this data. However, effects of boats, auroras, and additional temporal lights remain. The data has also been corrected for stray light in the summer months [23] which often experiences distorted values at higher latitudes, such as northern B.C., due to solar radiance persisting well into the night [34]. Due to this phenomenon, the summer months, specifically June, are of poorer quality even with the stray light correction [35]. Another natural seasonal variation is the influence of snow cover in winter. The high albedo of snow increases the backscatter of artificial light and creates exaggerated radiance values in snowy regions [36, 37], which includes most of the province excluding the coastal zone. To correct for the influence of snow we utilize the MODIS albedo daily 500 m dataset (MCD42A3 V6), specifically, the white sky albedo band which provides bihemispherical reflectance from the MODIS surface reflectance bands representing albedo under diffuse illumination conditions [38].

### Production data

Lumber production data were downloaded from Statistics Canada index #30031 [39] which provides monthly production volume and shipment volume over the duration of the study time period, January 2014, to December 2020. These data are also recorded separately for each of the three natural resource regions. Closure dates for individual mills were obtained from the British Columbia Mill Status Report compiled quarterly by the Province of B.C. Estimated production capacity for lumber mills was extracted from the Major Timber Processing Facilities Survey [40].

## Methods

### Pre-processing

VIIRS utilizes an on-board sensor calibration system to eliminate many of the natural inconsistencies present in the DMSP/OLS data [41]. It contains both an improved spatial resolution and an on-board calibration system which work together to substantially reduce the influences of saturation and blooming [22]. However, the increased radiometric resolution has been shown to detect nocturnal air glow from the Earth’s Ionosphere [42] which should be considered when interpreting VIIRS data. To address this issue, we apply a minimum threshold where any NTL value less than 1 nW/cm^2^/sr^-1^ is revalued to zero, based on the recommendation by Zhao et al. [35].

Both VIIRS NTL and MODIS albedo data were accessed using Google Earth Engine (GEE) [43] and the following procedure was completed within GEE to correct for seasonal influence of snow cover and background light. Code to access data provided in S2 File. First, the MODIS 500m daily white-sky albedo values were averaged into monthly composites to match the temporal frequency of the NTL data. The NTL values were then normalized for the influence of snow on NTL following the procedure of Man et al. [44] (Eq 1). Here, *ntl* represents the monthly VIIRS data and *albedo* represents the monthly white-sky albedo values.


$$Snow\;corrected\;radiance=ntl*(1.20148*0.051797^{albedo})$$ (1)

Lastly, the NTL data were normalized relative to the average background value of the surrounding area, to eliminate the influence of any long-term prevailing trends produced by the environment or external local events. Each mill was buffered by 500 m, representing the light from the mill, and then again by 5000 m to capture surrounding lights. When comparing changes in NTL overtime, the sum of lights (SOL) within the buffer zone were used as is standard practice [10, 22, 29, 45]. The SOL from the mill buffer were then subtracted from the SOL from the surrounding lights buffer to get the background SOL value. The mean background SOL over the time-series was calculated and any changes in the mill SOL were normalized relative to changes in the background value to minimize environmental fluctuations and the influence of urban areas surrounding the mills. This process allowed us to differentiate changes in the mill SOL from background SOL.

### Production capacity

To assess the relationship between lumber production and NTL radiance we first conducted a log-transformed regression between the mean radiance values observed over the seven-year time-series against the production capacity for each of the 122 identified lumber mills. Mills were stratified into 4 categories: city, closed, curtailed, and normal. The “city” category represents mills existing within population centers > 80,000 residents and largely consisted of mills from Metro Vancouver, Nanaimo, Kelowna, and Kamloops. Mills in the Closed and Curtailed category have been permanently closed or have had production/shifts curtailed since 2019 and were expected to produce less light. The “normal” category consists of all the mills that do not fall into any of the aforementioned categories.

We then assessed the capacity of NTL to observe mill closures at the individual mill level, specifically when a mill is shut down or has a production curtailment. We selected five pairs of mills that contain one mill which experienced a permanent closure during the assessment period and a nearby mill that has remained in operation. Two pairs were selected from the north, two from the south and one from the coastal region. The month of closure was determined for each pair directly from the B.C. Quarterly Mill reports [46] and the mean radiance of both mills was compared before, and after, closure using a Welch’s t-test. This test was selected as it is more robust than the student’s t-test and does not require the assumption of a constant variance. A time-series graph was also generated to visually assess the effect of the closure. This procedure was repeated for each of the 5 pairs.

Lastly, we examined if relationships exist between changes in mill SOL over the entire time-series with reported monthly production values, at monthly, seasonal, and annual reporting periods. For the coastal and southern regions, the SOL for every mill was totaled to generate a time-series with monthly intervals spanning all seven years and representing SOL of all lumber mills. The northern data were omitted from analysis due to multiple “zero” observations during the summer months due to stray light from solar radiance. We then conducted three regressions that compare production values to NTL SOL values annually, quarterly, and monthly (with a one-month lag in NTL data).

In order to effectively compare the association between time-series, each must be stationary to avoid spurious correlation [47]. First, the data was deseasonalized and then, using a vector autoregressive (VAR) model to select the optimal lag for each time-series, we assessed each one for stationarity using the Augmented Dickey-Fuller (ADF) approach [48] implemented with the “urca” package in R [49]. Non-stationary time-series, which have a stochastic trend, were then tested for co-integration using the Johansen method [50] and applying both the ‘trace” and “eigen” methods. Co-integration implies two time-series may move independently however their overall trends, measured by the average distance between them, remains relatively constant. This is present when a linear combination of two non-stationary time-series (integrated to order 1: *I(1))* results in a stationary time-series: *I(0)* [51] The presence of cointegration implies there is at least one unidirectional causal relationship between the time-series. To test for the significance of this influence, the Granger causality test was applied using the “lmtest” R package [52]. Stationarity is required when testing for Granger causality [53] therefore each time-series was differenced until stationarity was obtained [54]. Granger causality does not measure correlation but rather if the inclusion of one time-series (production) in a model, significantly enhances the predictive capability of other time-series (NTL) [53]. The Granger causality test was conducted in both directions as we expected production to influence NTL and not contrariwise.

## Results

**Fig 2:**
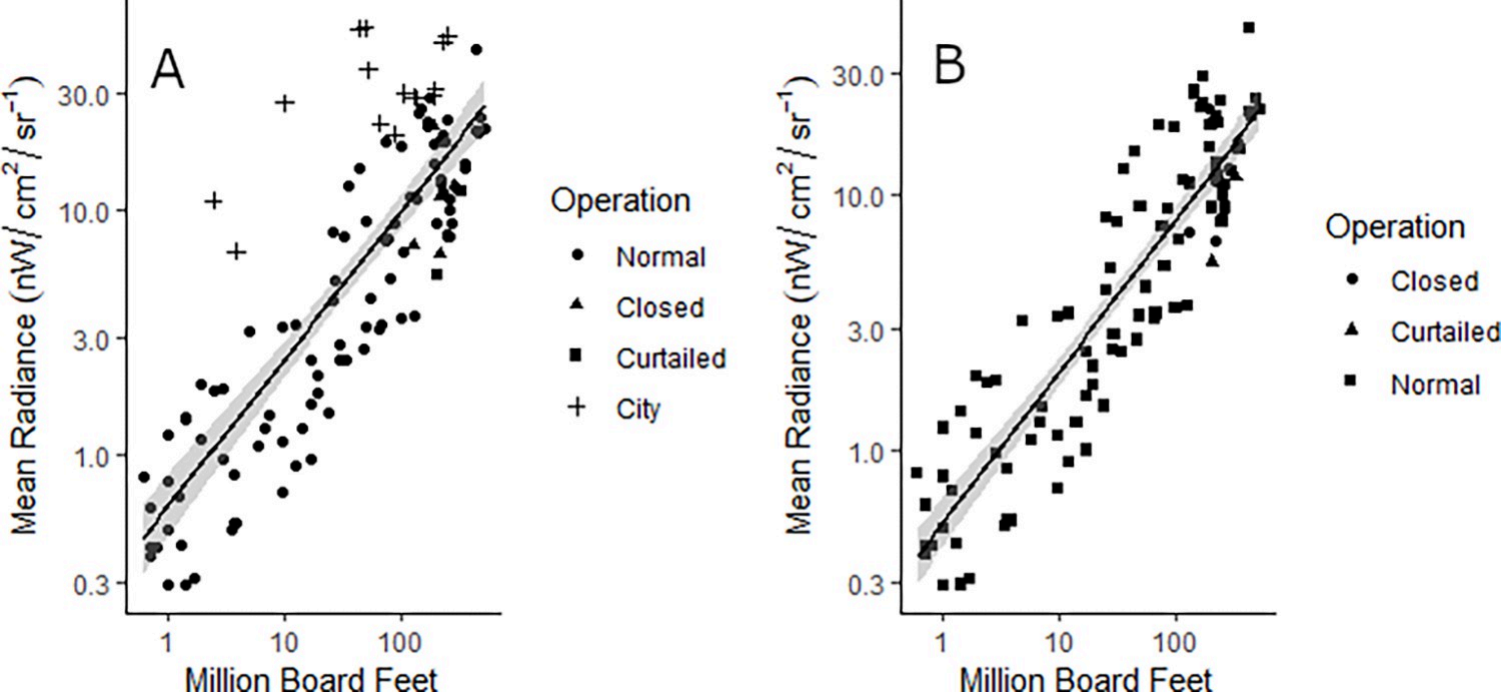
A) Regressions of lumber mill production (Million Boards Feet) against mean radiance of NTL. B) Regression excluding mills located directly within large populations centers (population >80,000).

Regression of production capacity against mean NTL radiance (Fig 2). Fig 2A shows all observed mills (n = 122) with a strong relationship and contains a strong correlation (R^2^ = 0.693, p < 0.01) with slight underestimation due to outliers that were brighter than expected. Most outliers are categorized as “city” (n = 14) suggesting there is a large quantity of external light spilling into the mill’s pixels, increasing the expected brightness. This influence is exemplified on the log scale where we had 3 mills producing less than 15 million board feet but still had a high mean NTL radiance. The “closed” and “curtailed” mills do not stand out as different from the rest of the population. Fig 2B represents the same regression excluding the “city” mills (n = 108) and presents a more consistent and significant relationship (R^2^ = 0.815, p < 0.01) between NTL radiance and lumber production capacity.

**Fig 3:**
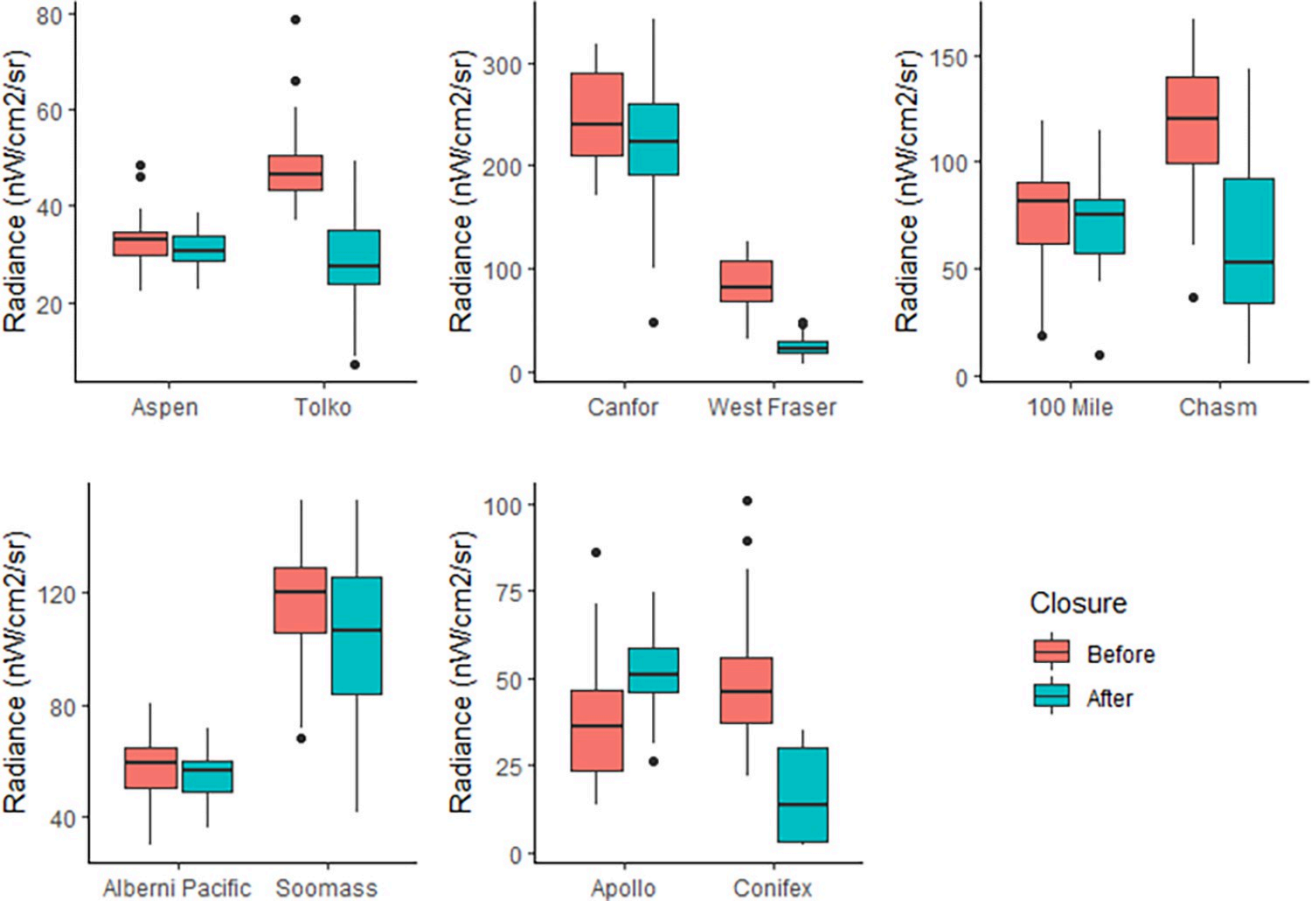
Change before and after closure date for each pair of mills A) Merritt, B) Houston, C) 100 Mile House, D) Port Alberni, E) Fort St. James.

When comparing mills that closed during the analysis period with their active neighbors, all five pairs of mills experienced a significant decrease in mean NTL after the designated closure date (Table 1). The Houston and 100 Mile House pairs showed decreases at the control mills (p = 0.623, p = 0.487) compared to significant decreases at the closed mills (p = <0.001) (Fig 3). These two pairs have the smallest sample sizes as their respective closure dates were close to the beginning and end of the time-series. The control mill for the Fort St. James pair experienced a significant increase in mean NTL (p = 0.012) while the closed mill significantly decreased to near zero (p = <0.01). Both the Port Alberni and Merritt pairs, which have the most balanced population sizes, experienced a significant drop for the closure mills (p = <0.01). However, the control mill for both these pairs saw a notable drop as well, but not quite significant (p = 0.098, p = 0.106) at our given alpha (α = 0.05). Table 1 also suggests that the northernmost pairs saw the most drastic differences between the control and closure mills.

**Table 1:** Results of Welch’s t-tests comparing NTL response of paired mills before and after mill closure date.

Mill Location	Status	Company	Region	n before	n after	p-value
Merritt	Open	Aspen Planers	South	37	47	0.106
Merritt	Closed	Tolko	South	37	47	<0.01***
Houston	Open	Canfor	North	10	74	0.6237
Houston	Closed	West Fraser	North	10	74	<0.01***
100 Mile House	Closed	West Fraser (Chasm)	South	67	17	<0.01***
100 Mile House	Open	West Fraser	South	67	17	0.4869
Fort St James	Open	Apollo Forest Products	North	66	18	<0.01***
Fort St James	Closed	Conifex	North	66	18	0.0121**
Port Alberni (Soomass)	Closed	Western Forest Products	Coast	45	39	<0.01***
Port Alberni (Pacific)	Open	Western Forest Products	Coast	45	39	0.098
Merritt	Open	Aspen Planers	South	37	47	0.106
Merritt	Closed	Tolko	South	37	47	<0.01***

**Fig 4:**
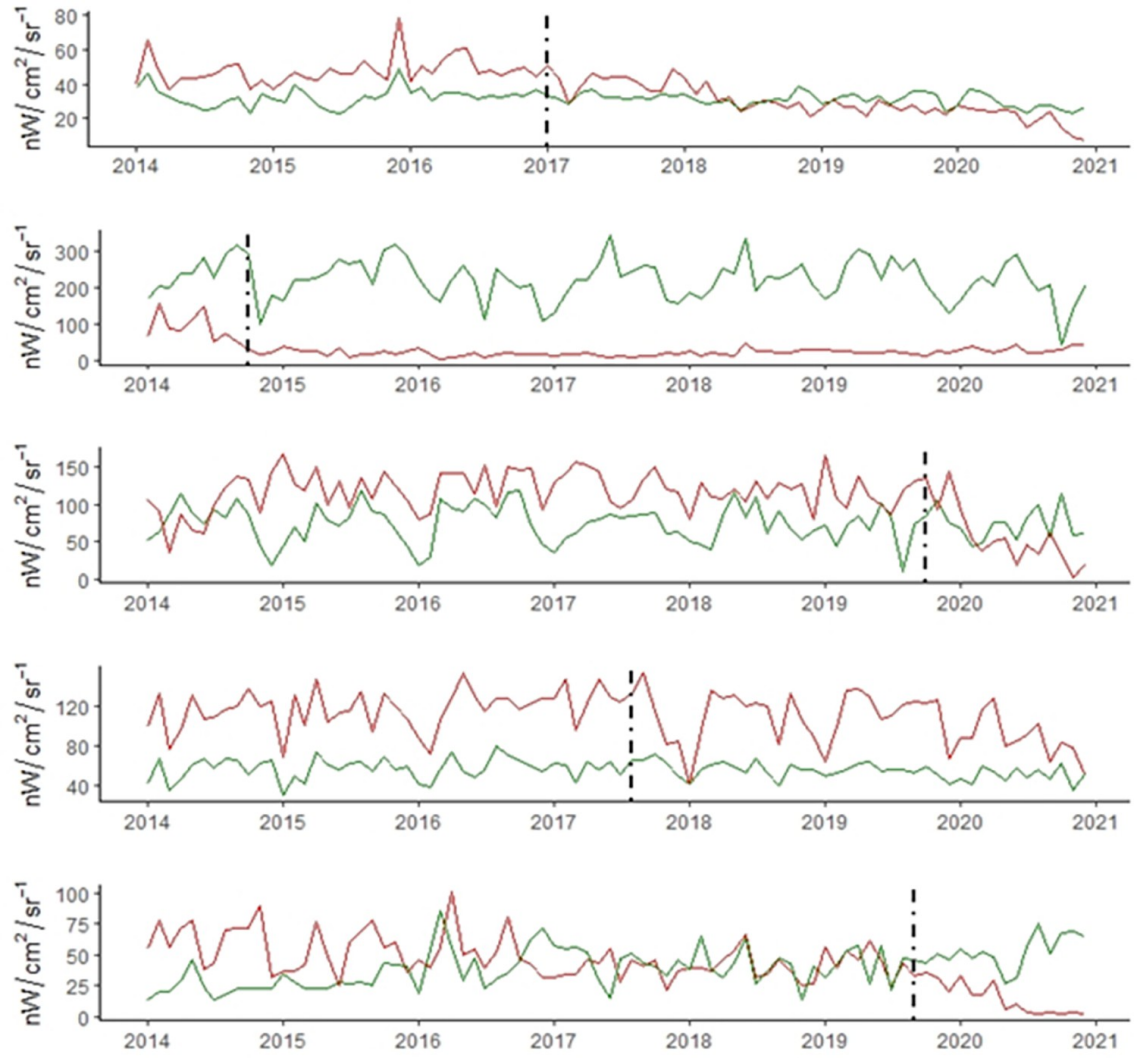
Time-series indicating mean NTL for each pair of mills, operational mill in green, closed mill in red and closure date as a vertical black line. A) Merritt, B) Houston, C) 100 Mile House, D) Port Alberni, E) Fort St. James.

Analysis of the time-series surrounding the closure date for each pair (Fig 4) displays a delayed response between closure date and reduction of mean NTL. There appears to be 6-12-month delay between closure and a substantial decrease in mean NTL. The Merritt, 100 Mile House, and Fort St. James pairs all have their control mill displaying a lower average consistently until the closure date where this trend reverses and the control mill holds steady while the closure affected mill decreases. The more remote closed mills (Houston, 100 Mile House and Fort St. James) eventually drop to near zero. In contrast, the Port Alberni and Merritt mills which are nestled within their respective population centers, experience a drop but do not reach zero. Notably, both these pairs appear to be experiencing a general downward trend over the entire time-series.

**Fig 5:**
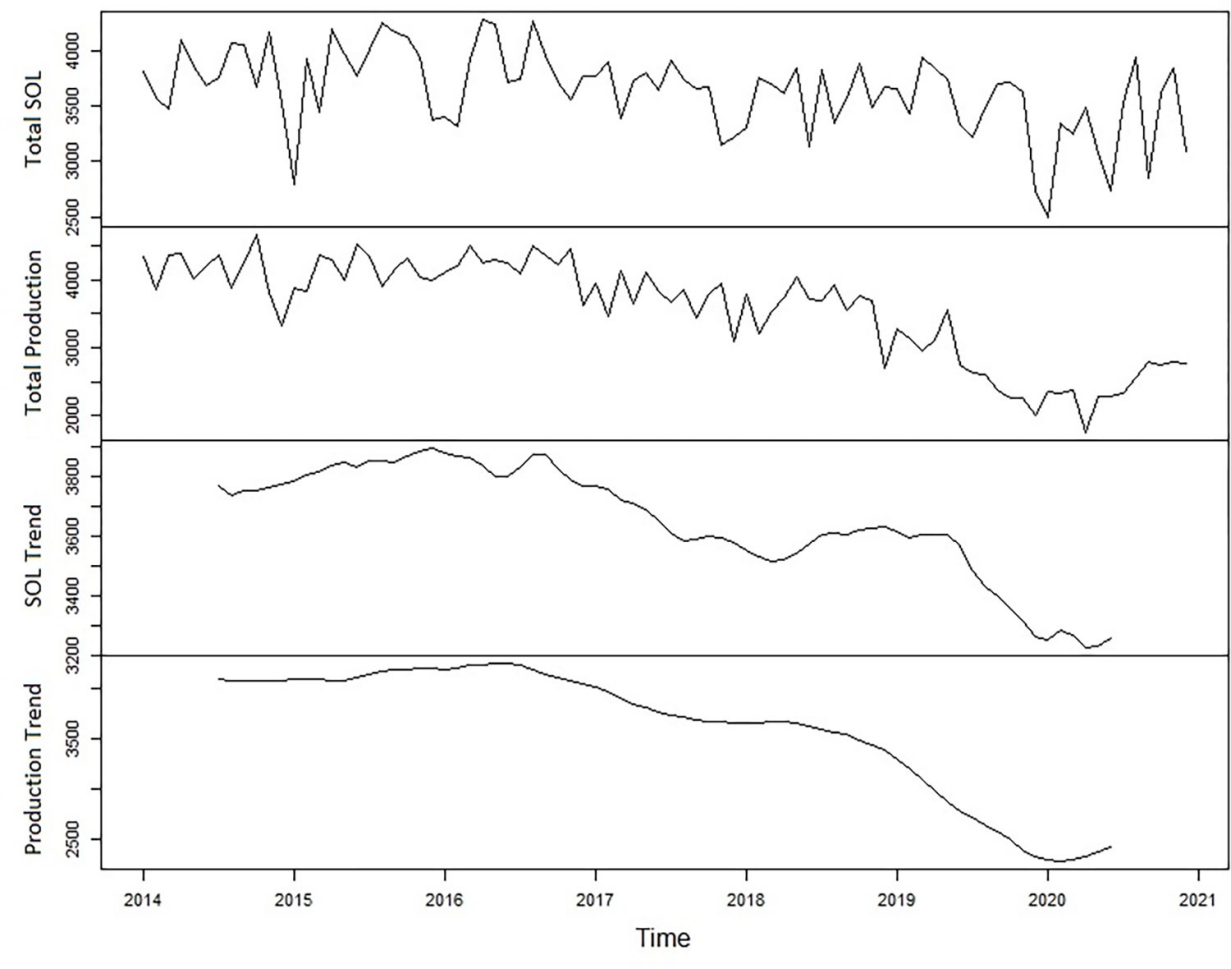
Total and raw and trend data of production and SOL for the combination of the south and coastal regions.

The raw time-series and associated trend measuring total production and SOL for all mills in the coastal and southern region are displayed in Fig 5. The two time-series are not highly correlated over the study period but tend to have similar peaks and troughs and both clearly exhibits a downward trend, with peaks in 2016 and in 2018. The largest deviations appear in the summer months where the data is known to be less reliable. The long-term trend between the two is very similar (Fig 5) with SOL appearing to lag 1–2 months behind production. The notable downward trends visible in Fig 5 are supported by the results of the ADF test which both accept the null hypothesis of unit-root presence (α = 0.05), suggesting both are non-stationary time-series. The constructed VAR models suggested lags of 3 and 2 for NTL and production respectively. Using these results, we were able to meet the assumptions for the Johansen co-integration test which rejected the null hypothesis of there being zero co-integrated time-series. With co-integration confirmed, stationarity of both time-series was achieved after taking the first difference of each time-series and re-applying the ADF test (α = 0.05). When the stationary time-series’ were tested for Granger causality, including the production time-series and its associated lags significantly improved the prediction of future NTL values (*p* = 0.013) whereas the inclusion of the NTL values did not improve the prediction of future lumber production values (*p* = 0.811).

**Fig 6:**
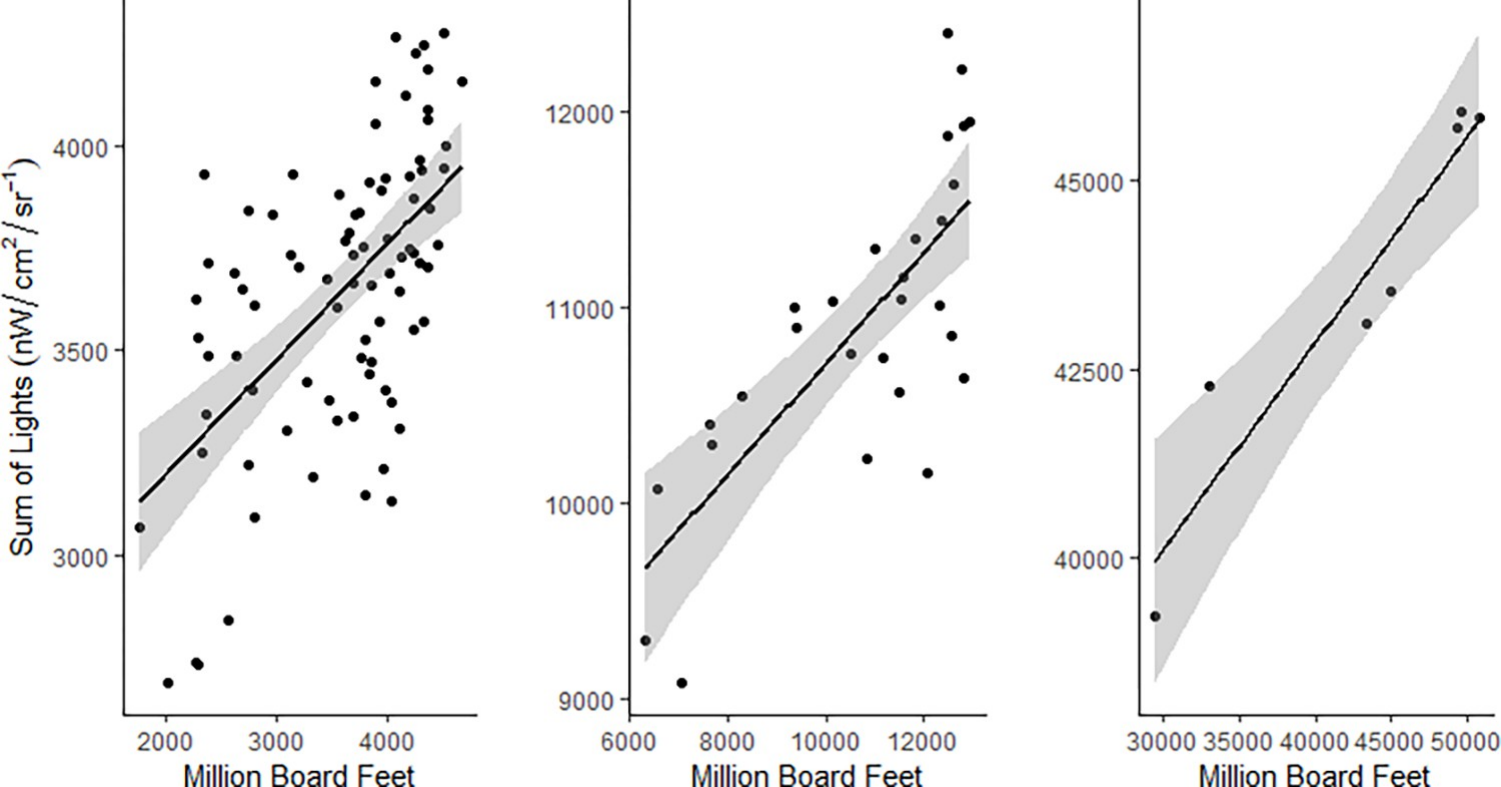
Regression of sum of NTL lights against lumber production at varying temporal scales. A) Monthly, B) Quarterly, C) Yearly. Shaded grey area represents the 95% confidence intervals.

This association between lumber production and NTL SOL is further supported by the regression graphs (Fig 6) which demonstrate a linear relationship between production and SOL at varying temporal scales. At a monthly scale, production and one-month lagged SOL display a trend but also a notable amount of variation (R^2^ = 0.332, n = 84, p = < 0.01). There does not appear to be a direct correlation between the two however, they do appear to be positively related. This relationship is strengthened when observed over a coarser temporal scale such as quarterly (R^2^ = 0.581, n = 28, p = < 0.01). The trend is considerably stronger at the quarterly level with much less variation, except at the extremes. Lastly, the yearly model displays the strongest relationship (R^2^ = 0.901, n = 7, p = < 0.01) with all observations falling within the confidence intervals (α = 0.05). The yearly model is limited by the small sample size as only seven years of data have been recorded.

## Discussion

In this study we characterize the relationship between lumber mill production and detected radiance from the NTL dataset. We demonstrate the ability of NTL to quantify production capacity at lumber mills as well as detect mill closures. We also examined the temporal relationship between lumber production and NTL. Results suggest that a strong, positive, logarithmic relationship exists between mean NTL and mill production capacity which reinforces the concept that brighter NTL is positively related to greater measures of economic wealth, GDP and anthropogenic activity [55–57]. Many of these high-production mills have larger facilities that require greater illumination for their industrial activity as they run nightshifts and have considerably more freight traffic, which has previously been quantified by NTL [25]. However, even with the VIIRS updated on-board calibration system, blooming can still be influential at fine spatial scales, especially in large urban centers [57] This effect is due to strong external urban light sources influencing the mill pixels and establishing a base level of illumination that results in overestimation as demonstrated by Huang et al. [58]. Our results support this contention that mills located in large urban centers, are disproportionally brighter. This matches previous findings regarding identifying light pollution in urban centers [15]. When these mills are removed from the population the R^2^ value increases considerably, suggesting they substantially deviate from the trend. This is especially true for relatively smaller mills that are located within urban industrial parks, which coincide with our greatest outliers in this study.

Analysis of the five paired mills further demonstrates the ability of NTL data to differentiate production levels, including on a fine spatial scale. Within each pair of mills over the observed time period, the closed mill showed a significant decrease in NTL. This highlights the ability of NTL to detect spatio-temporal patterns in lumber mill light pollution, similar to patterns found for other industrial and urban activities [41]. Despite the limited number of observations before/after the closure date for the Houston, 100 Mile House and Fort St. James pairs, the time-series graphs clearly demonstrate a downward trend after closure. The decrease in NTL is a delayed response which is conceivable given that it is unlikely for the mill to cease operation immediately. Mills are likely emptied slowly of all remaining goods and then dismantled requiring considerable freight logistics [59] which may be influencing this delayed decrease in NTL. Within each mill pairing, the active mills remained nearly unchanged and experienced only small fluctuations, likely associated with environmental patterns.

Alternatively, the Merritt and Port Alberni mills affected by a closure significantly decreased but considerable decreases were seen for the active mills as well. One explanation might be that the mills are close enough to each other that the closed mill reduced external light enough to influence the SOL for the active one. This is conceivable given that both pairs are located ~ 1km from each other. Another consideration is that both mills are located within their respective townsites and if the town saw an overall decrease in NTL, this would be reflected at the mill. Both Merritt and Port Alberni are forestry towns that have experienced stagnant or declining populations over the study period [60]. The mills are also located in the older parts of the towns, and both are experiencing concentrated growth focused in newly developed areas. Hayter and Nieweler [61] outline this phenomenon for Port Alberni specifically which effectively transfers known NTL proxies such as, wealth, GDP, and anthropogenic actively away from older areas of town where the mills are, potentially explaining a decline in NTL.

Given the success of NTL to spatially detect production, the temporal results were more ambiguous. Specifically, time-series correlation was strongest at the annual and quarterly time scales. An undoubtable influence of this temporal discrepancy is the 1-2-month lag between production and NTL. When compared over a small time period such as 1 month, this lag may be spread between two time periods. When scaled up to a quarterly resolution, much of the variation between time periods is eliminated and a stronger relationship established. This trend continues at the annual scale where there is a clear relationship with minimal variation demonstrating excellent potential to be used as a proxy for lumber production. This coincides with a recent study in China that was successfully able to use annual NTL as a proxy for freight traffic [62]. However, for our study there are only seven years provided for the annual measurements resulting in a small *n*. As such, the true annual relationship may not be as strong as presented but given the other temporal scales, it is very likely stronger than the quarterly result, which is still substantial. Studies utilizing the longer historical data of the DMPS/OLS nighttime lights are suggestive of strong annual trends [8, 15]. As the NTL data set continues to grow for the foreseeable future, more annual measurements will become available, and a more robust model can be built.

Regardless of temporal scale, some degree of association exists between lumber production and NTL as they proved to be co-integrated and have unidirectional Granger causality. Previous literature has found co-integrated relationships between NTL and population [10], GDP [63] and electricity consumption [64]. All these measures are positively related to commodity production [65] so a similar relationship is to be expected. Our findings of the causal relationship between lumber production and NTL being unidirectional also supports this idea. Given NTL is measuring artificial light it should be driven by anthropogenic activity and not the opposite.

The 1-2-month lag also highlights the delay between NTL measurements and reported production. Theoretically, the relationship should be captured in real-time [66] but this is not the case. One consideration is the presence of lags in production data reporting as well as stochasticity related to inter-month production. Another is that the NTL activity measures are not only capturing production, but rather a mixture including inter-mill lumber transportation and export shipments since NTL is known to capture freight and industrial activities [24, 62]. These activities would likely not be concurrent with production and would contribute to measurement lags. Regardless, NTL has been shown to parallel recent industrial production trends [5] similar to our findings, thus demonstrating its capability to be used as a long-term causal proxy for production. It would have been ideal to compare individual mill SOL with production, however, production data was unavailable on a mill-by-mill basis. Our results indicate that despite this, we should be able to use NTL to measure relative production without reported data.

Since no production data is available on a mill-by-mill basis, a linear model using historical NTL SOL data could be used to predict lumber production for individual mills or groups of mills as soon as imagery becomes available. Specifically, the quarterly model reduces noise to a reliable level and could provide an early warning system for production trends that heavily influence harvest and employment numbers. It is also possible that the quarterly model may even perform better on an individual mill level. This strategy was tested on the quarterly data by building a linear regression model and utilizing different cross-validation approaches to test for accuracy. When using 70% of the data for training the model and reserving 30% for validation, R^2^ values ranged between 0.54–0.79. This is suggestive of a strong relationship and presents forest industrial interests with a feasible method to predict production trends without reported data.

Despite the recent technological advances, there are limitations associated with this study and the usage of NTL generally. Reliable NTL measurements are limited by latitude, as elongated summer days at northern latitudes cause stray light to interfere with NTL measurements. This effect was noticeable in our study as much of the northern region (above 54°N) displayed extreme radiance values during the months of May, June, and July and had to be omitted from the analysis. The stray light correction algorithm [67] implemented into the NTL data set allowed our analysis to be conducted in the southern half of British Columbia, which previously consisted of many months with no reliable observations but could not reliably account for the impact farther north. Another limitation regarding the spatial application of NTL is the lack of ability to differentiate fluctuations of lights in urban areas. Our method of normalizing in relation to the background values appears to have worked to an extent but would likely be less effective in dense urban centers. Comparing NTL values at fine spatial scales is considerably better suited for rural or suburban areas where there are fewer external variables contributing to light pollution and pixel spillover.

Fluctuations and long-term trends gathered from NTL exemplify how discernable industrial-level economic variations are, even from space. The ability to measure this variation provides a unique and openly available vantage point into the systematic framework of forestry and potentially other industries. While previous studies have linked economic growth and human wealth levels to NTL [55], this study provides an alternative viewpoint focusing solely on the industrial angle. For “boom and bust” industries like forestry that are often located in small, resource-based towns, industrial production has a profound effect on local economies and well-being. Future opportunities exist to expand on NTL measurements and integrate knowledge to characterize the patterns associated with the resource-based town economic trends. In North America, most of these towns are experiencing economic decline [61, 68]. Examining past conditions coupled with NTL could be used to establish robust, near real-time models to help communities respond to, react to and prevent the extreme fluctuations associated with resource-based communities. Additionally, future applications of NTL could be used to characterize the recent unprecedented surge in lumber prices associated with the COVID-19 pandemic [69] that has put pressure on lumber manufacturers as they struggle to meet demand.

## Conclusion

NTL data derived from the VIIRS DNB sensor is a useful measurement of anthropogenic development and economic activity. We demonstrate the capability of NTL to measure lumber production on both a spatial and temporal scale over a seven-year period in British Columbia, Canada. Specifically, we identify a strong logarithmic relationship between mean NTL radiance and production capacity of lumber mills. This relationship is reinforced by comparing pairs of nearby mills where one experienced a permanent closure and the other remained active. The NTL time-series could significantly differentiate between open and closed mills for all five pairs. Lastly, significant co-integration was found over time between two time-series describing the monthly NTL and monthly lumber production. Linear models comparing the two annually demonstrated a strong correlation that weakened with increasing temporal resolution. These models present an opportunity to predict lumber production in the absence of reported data. This would allow for prediction at individual and company level without the delay of reported statistics. Additionally, lumber production was shown to Granger cause NTL, further suggesting a causal relationship between them. The evidence provided by this study suggests that the SOL from lumber mills could reasonably be used as a long-term proxy for lumber production and potentially other industrial commodities. As the NTL time-series continues to lengthen, more data will become available allowing for more robust models and measurements to further characterize the relationship between NTL and forestry production.

## Supporting information

S1 File Lumber mills used in study. Information about the name, size and locations of each lumber mill used in this study.
(ZIP)

S2 File NTL satellite extraction code. This code was used to collect the VIIRS and MODIS input modelling variables. The data is freely available and can be extracted by pasting the following code into the Google Earth Engine code editor.
(DOCX)
