# Supplementary material for: Measuring industrial lumber production using nighttime lights: A focus study on lumber mills in British Columbia, Canada
Source: PLoS One. 2022 Sep 13;17(9):e0273740. doi: 10.1371/journal.pone.0273740 (PMC9469976; doi:10.1371/journal.pone.0273740)
Supplement: S1 File — Information about the name, size and locations of each lumber mill used in this study. (ZIP) [file pone.0273740.s001.zip › GSR_TMBR_PRCSSING_FAC_SV.html]

Data Catalogue **We're sorry but this site doesn't work properly without JavaScript enabled. Please enable it to continue.**
