# Supplementary material for: Measuring industrial lumber production using nighttime lights: A focus study on lumber mills in British Columbia, Canada
Source: PLoS One. 2022 Sep 13;17(9):e0273740. doi: 10.1371/journal.pone.0273740 (PMC9469976; doi:10.1371/journal.pone.0273740)
Supplement: S2 File — This code was used to collect the VIIRS and MODIS input modelling variables. The data is freely available and can be extracted by pasting the following code into the Google Earth Engine code editor. (DOCX) [file pone.0273740.s002.docx]

## Google Earth Engine code

The following code was used to collect the VIIRS and MODIS input modelling variables. The data is freely available, and can be extracted by pasting the following code into the Google Earth Engine code editor. (https://code.earthengine.google.com/ ).

| /////////////////////////////////////////////////////////////////  //data setup  /////////////////////////////////////////////////////////////////  // Shapefile for mill locations (retrivied from BC gov)  var Mill_locations = ee.FeatureCollection(mill_BC_gov);//must define mill shapefile as asset in GEE  // Select just lumber mills  var LBR = ee.Filter.inList('PRODUCT_CD', ['LBR']);  var mills = Mill_locations.filter(LBR);  /////////////////////////////////////////////////////////////////  //Nightime lights data retrive  /////////////////////////////////////////////////////////////////  //retrieve VIIRS Nightlights data  var dataset = ee.ImageCollection('NOAA/VIIRS/DNB/MONTHLY_V1/VCMSLCFG')  .filter(ee.Filter.date('2014-01-01', '2021-05-28'));  //select radiance  var ts = dataset.select('avg_rad');  //set visualization parameters  var nighttimeVis = {min: 1.0, max: 80};  // Set Date range for assessment  var early = ee.ImageCollection(("NOAA/VIIRS/DNB/MONTHLY_V1/VCMCFG"))  .filter(ee.Filter.date('2012-04-01', '2020-05-01'));  /////////////////////////////////////////////////////////////////  //Snowcover data retrive  /////////////////////////////////////////////////////////////////  ///Snowcover and albedo  var predataset = ee.ImageCollection('MODIS/006/MCD43A3')  .filter(ee.Filter.date('2014-01-01', '2021-05-29'));  var snowcover = predataset.select('Albedo_WSA_vis');  /// Change snowcover from daily to monthly  var month_mean = ee.List.sequence(0, 89).map(function(n) { // .sequence: number of years from starting year to present  var start = ee.Date('2014-01-01').advance(n, 'month'); // Starting date  var end = start.advance(1, 'month'); // Step by each iteration  return ee.ImageCollection("MODIS/006/MCD43A3")  .select('Albedo_WSA_vis')  .filterDate(start, end)  .mean()  .set('system:time_start', start.millis());  });  /////////////////////////////////////////////////////////////////  //combine nightlights and snow to same temporal scale  /////////////////////////////////////////////////////////////////  var mod1 = ts;  var mod2 = month_mean;  var filter = ee.Filter.equals({  leftField: 'system:time_start',  rightField: 'system:time_start'  });  // Create the join.  var simpleJoin = ee.Join.inner();  // Inner join  var innerJoin = ee.ImageCollection(simpleJoin.apply(mod1, mod2, filter));  //Apply  var joined = innerJoin.map(function(feature) {  return ee.Image.cat(feature.get('primary'), feature.get('secondary'));  });  /////////////////////////////////////////////////////////////////  //Make Snow corrected band for NTL  /////////////////////////////////////////////////////////////////  var addcor = function(image) {  var snowcor = image.expression(  'RAD* 1.201483* 0.051797**(WSA/1000)', {  'RAD': image.select('avg_rad'),  'WSA': image.select('Albedo_WSA_vis')})    return image.addBands(snowcor);  };  // Second, apply function over Collection  var snow_correct = joined.map(addcor);  //isoalte adjusted time-series and apply  var snowy = snow_correct  .filter(ee.Filter.date('2014-01-01', '2021-05-28'));  var adjusted = snowy.select('avg_rad_1');  //add to map  Map.addLayer(adjusted, nighttimeVis, 'Radiance');  Map.addLayer(mills,{color:'FF0000'}, 'Timber locations');  //make buffer function  var bufferBy = function(size) {  return function(feature) {  return feature.buffer(size);  };  };  //500m buffer around mills  var buffmill = mills.map(bufferBy(500));  //larger buffer around mills for normalization  var buffallmill = mills.map(bufferBy(5000));  //Map.centerObject(testPoint, 10)  /////////////////////////////////////////////////////////////////  //Print charts  /////////////////////////////////////////////////////////////////  var mill_light = ui.Chart.image.series({  imageCollection: adjusted,  region: buffmill,  scale: 500,  reducer: ee.Reducer.sum(),//could change to mean  }).setOptions({  interpolateNulls: true,  lineWidth: 1,  pointSize: 3,  title: 'Sum of monthly mill radiance for 500m around all mills',  vAxis: {title: 'Radiance'},  hAxis: {title: 'Date', format: 'YYYY-MMM', gridlines: {count: 12}},    });  print(mill_light);  var normalize_background = ui.Chart.image.series({  imageCollection: adjusted,  region: buffallmill,  scale: 500,  reducer: ee.Reducer.sum(),  }).setOptions({  interpolateNulls: true,  lineWidth: 1,  pointSize: 3,  title: 'Sum of monthly mill radiance for 5000m around all mills',  vAxis: {title: 'Radiance'},  hAxis: {title: 'Date', format: 'YYYY-MMM', gridlines: {count: 12}},    });  print(normalize_background);  /////////////////////////////////////////////////////////////////  //END SCRIPT  ///////////////////////////////////////////////////////////////// |
| --- |
